# Factors associated with non-adherence to antiretroviral therapy among HIV-infected adolescents aged 15-19 years: a snapshot from the Mother and Child Center in Yaounde, Cameroon

**DOI:** 10.11604/pamj.2021.39.154.27623

**Published:** 2021-06-29

**Authors:** Martial Wandji Lantche, Joseph Fokam, Anne Jocelyne Nguemedyam Cheudjui, Jules Brice Mbougua Tchatchueng, Thierry Serge Joël Noumsi, Francis Ndongo Ateba, Paul Ndombo Koki, Clotaire Serge Billong

**Affiliations:** 1 Department of Epidemiology, School of Health Sciences, Catholic University for Central Africa, Yaounde, Cameroon,; 2 Mother and Child Center, Chantal Biya Foundation, Yaounde, Cameroon,; 3 Department of Medical Laboratory Sciences, Faculty of Health Sciences, University of Buea, Buea, Cameroon,; 4 Virology Laboratory, Chantal Biya International Reference Center for research on HIV/AIDS prevention and management, Yaounde, Cameroon,; 5 Department of Microbiology, Heamatology, Immunology, Parasitology and Infectious Diseases, Faculty of Medicine and Biomedical Sciences, University of Yaounde I, Yaounde, Cameroon,; 6 Department of Gynecology and Obstetrics, University of Yaounde I, Yaounde, Cameroon,; 7 Department of Epidemiology, Pasteur Center of Cameroon, Yaounde, Cameroon,; 8 Department of Public Health, Faculty of Medicine and Biomedical Sciences, University of Ngaoundere, Ngaoundere, Cameroon,; 9 Department of Pediatrics, Faculty of Medicine and Biomedical Sciences, University of Yaounde I, Yaounde, Cameroon,; 10 Department of Public Health, Faculty of Medicine and Biomedical Sciences, University of Yaounde I, Yaounde, Cameroon

**Keywords:** Factors associated, non-adherence, adolescents, Yaounde, Cameroon

## Abstract

**Introduction:**

non-adherence to antiretroviral therapy (ART) constitutes the main cause of therapeutic failure among HIV-infected adolescents, especially in the aged group 15 to 19 years. We aimed to determine factors associated with this non-adherence in this specific population.

**Methods:**

we conducted a cross-sectional study at the Mother and Child Center in Yaounde from August to October 2018. Delayed clinic appointment was referred to as defaulters. Non-adherence was measured during the 3 days preceding inclusion by self-reported method following quantitative (missing dosage of ART), qualitative (ART taken with a delay of more than 2 hours) and combined measure. A threshold of non-adherence > 20% was considered high, with p<0.05 statistically significant.

**Results:**

overall, 195 out of 251 (77.7%) eligible adolescents were included, of which 56.9% were girls (sex-ratio = 4/3). The mean age was 16.8 ± 1.5 years. The rate of defaulters was 21.0%. Following quantitative approach, 33.8% were non-adherent. Using combined approach, we had 41.0%. This non-adherence was associated with duration on ART > 5 years (adjusted Odds Ratio [aOR]: 2.33;95% Confidence Interval [CI]: 1.08-5.00; p:0.030), defaulters (aOR: 2.56;95% CI: 1.12-5.82; p:0.025) and HIV Viral Load (VL) ≥ 40 copies/ml (aOR: 0.42; 95% CI: 0.21-0.83; p:0.013).

**Conclusion:**

at this reference pediatric center, 4 out of 10 adolescents aged 15-19 years on ART are non-adherent, driven by missing dosage of drug intake. Strategies for enhanced adherence for late age adolescents are therefore warranted, by prioritizing interventions on defaulters and duration on ART > 5 years.

## Introduction

Non-adherence to ART is referred to as missing dosage of ART (quantitative measure) or ART taken with a delay of more than 2 hours (qualitative measure) or both (combined quantitative and qualitative measure) during the 3 days preceding inclusion by self-reported method. Acquired Immunodeficiency Syndrome (AIDS) is the second cause of mortality in adolescents worldwide and the first in Africa [1, 2]. HIV-related deaths have decreased in all age groups between 2000 and 2015, except for adolescents where mortality rate has doubled (> 50%) from 18,000 to 41,000 [3, 4]. Worldwide, non-adherence to ART constitutes the main cause of therapeutic failure [5-7] and is prevalent in 64% among HIV-infected adolescents on ART [8]. A rate of adherence of at least 95% is required to maintain long-term therapeutic success and limit drug resistance [9].

A study conducted in Douala among HIV-infected adults at Laquintinie Hospital revealed that 49% were non-adherence [10]. In this study, factors such as widows, excitants consumption and opportunistic infections were associated with non-adherence. At the Mother and Child Center (MCC) in Yaounde, a cross-sectional study about factors associated with adherence to ART in children under 15 years reported that 16.4% were non-adherence [11]. This non-adherence was associated with age, difficulties in receiving medication at the pharmacy, non-biological parents. At the Dschang District Hospital, a cross-sectional study found that the rate of non-adherence was 19.8% [12]. In the Center Region of Cameroon, a cross-sectional study conducted among 401 adolescents receiving ART revealed that living beyond 5 km from the heath facility, taking medications in the same service with adults and managed at a rural health facility without regular counseling were associated with non-adherence [13].

The aim of this study was to determine factors associated with non-adherence to ART among HIV-infected adolescents aged 15 to 19 years and followed-up at the MCC reference pediatric center in Yaounde. Specifically, this study aimed at measuring the association between sociodemographic, clinical, biologic, enhance adherence factors and non-adherence to ART.

## Methods

**Study design:** we carried out a cross-sectional study to determine factors associated with non-adherence to ART among adolescents receiving ART at the MCC from August to October 2018. The following points justify the choice of this study site: (a) the MCC is the first center of excellence in Cameroon specialized in monitoring Adolescents Living with HIV (ALWHIV) on ART; (b) it is the health facility having the highest number of ALWHIV on ART in Cameroon. Study population was adolescents aged 15-19 years who came for their routine follow-up.

**Sampling:** we used a consecutive sampling. Adolescents were included based on the following criteria: (a) aged 15-19 years; (b) on ART for at least 6 months; (c) registered for ART monitoring in the study site; (d) HIV-disclosure status completed and (e) who have provided their consent/assent. The minimum sample size was estimated using the formula z^2^p (1-p)/e^2^; Where n= sample size, z= 95% confidence interval= 1.96, p= proportion of non-adherent= 64% [8] and e= sampling error margin= 7%. Thus, the minimum sample size is n= 181.

**Data collection:** after pretesting the questionnaire, data collection was done by the principal investigator during routine clinic attendance of adolescents in the study site. Each adolescent was seen only once. The average time for completing a questionnaire was 20 minutes. About 3 questionnaires were completing every day from Monday to Saturday. Non-adherence was measured during the 3 days preceding inclusion by self-reported method following 3 approaches: (a) quantitative (missing dosage of ART); (b) qualitative (ART taken with a delay of more than 2 hours) and (c) mixed (quantitative and qualitative). These approaches were already used in other studies [14-16]. A threshold of ART non-adherence > 20% was considered high. Two questions were used to determine the proportion of non-adherent participants. On the one hand, the question: during the past three days, have you missed a dose of your ART? To which people could answer “yes” or “no”, and on the other hand the question: during the last three days, have you delayed more than 2 hours a dose of your ART? To which people could answer “yes” or “no”. People were considered non-adherent if they had “missed a dose of their treatment” or “delayed their treatment for more than 2 hours” at least once during the 3 days preceding their inclusion. Those who proposed other responses were classified as adherent. Delayed clinic appointment was referred to as defaulters. HIV knowledge was assessed on 15 points and participants scoring at least 12/15 were considered having good knowledge. Otherwise, they were considered having poor knowledge.

**Data analysis:** statistic tests were performed to compare the characteristics of adherents and non-adherents. Quantitative variables were converted into qualitative variables that were analyzed using the chi-square test. Logistic regression was used to estimate Odds-Ratios and their confidence interval in univariate and multivariate analyzes. Associated variables in univariate analyzes with a significance level < 0.20 were considered eligible in the multivariate model, with the exception of factors that could be clearly considered as a consequence Variables whose response modalities discriminated less than 5% of the sample were not considered eligible for the initial model. Variables independently associated with a value of p <0.05 were retained in the final model. SPSS version 21 software was used for all statistical analyzes. All p-values < 0.05 were considered statistically significant.

**Ethical approval and consent to participate:** the protocol of this study was approved by the Institutional Ethics Committee of the Catholic University of Central Africa (N° 2018/0775/CEIRSH/ESS/MSP). After obtaining consent of parents/guardians and assent of adolescents, a standardized questionnaire was used to collect sociodemographic, clinical, laboratory and enhance adherence strategies data.

**Data confidentiality:** the confidentiality of these data was managed by a system of anonymity based on a code (CE/O8/M/A/X, where: CE= Center region, 08= MCC-CBF, M= Month of registration of the participant in the ward, A= Year of registration, X= Registration number in the month). The completed questionnaires were kept in a locked drawer in the office of the principal investigator. Data treated were stored in a digital file of which access was only possible through the use of the investigator´s password.

## Results

**Figure 1:**
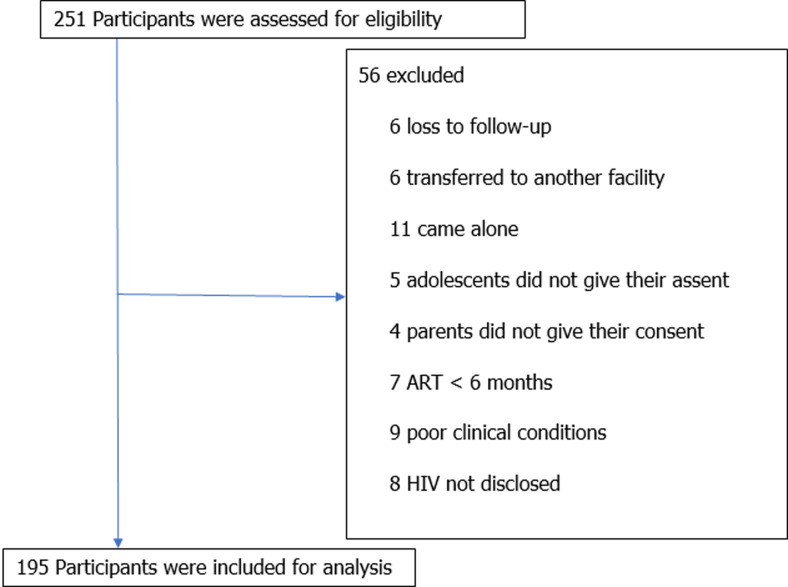
participant’s eligibility assessment and analysis, Mother and Child Center in Yaounde, August to October 2018

**Participants:** of the of 251 eligible participants, 195 were included (77.7%) (Figure 1). Among the 56 excluded, we had: (a) 6 loss to follow-up, (b) 6 transferred to another facility, (c) 11 came alone, (d) 5 adolescents refused to give their assent, (e) 4 parents refused to give their consent, (f) 7 on ART < 6 months, (g) 9 poor clinical conditions, (h) 8 HIV not disclosed.

**Descriptive data:** the proportion of non-adherence to ART was 41.0% (80/195) for combined measure, with 33.8% (66/195) quantitative and 7.2% (14/195) qualitative (Table 1). The mean age was 16.8 ± 1.5 years and 56.9% (111/195) were girls (sex-ratio = 4/3). About 16.4% (32/195) were living out of Yaounde and 1 adolescent out of 10 (9.2%) were not schooling. Among those who were schooling, 9.2% (18/195) were still in primary school. Also, we found that 21.5% (42/195) were sexually active and 29.2% (57/195) were taking alcohol (Table 1). About 21.0% (41/195) of participants were orphans of both parents. Vertical transmission (90.2%) was the most represented. Asymptomatic participants (93.3%) were the highest. Few participants (21.0%) were defaulters. Likewise, 26.7% (52/195) were on second line regimen. Almost all adolescents (92.8%) were on once-a-day medication. Also, 72.8% (142/195) were under Efavirenz regimen and 42.1% (82/195) had CD4 count ≤ 500 cells/mm^3^ (Table 1). Almost 50.0% (94/195) of adolescents knew their HIV status after 13 years. This disclosure was done by health personnel in 73.3% (143/195) of cases. The hour of drug intake was reminded by 13.8% (27/195) of parents/guardians. Transition preparation from pediatric to adult clinic was not yet started for many cases (70.3%). Moreover, 47.2% (92/195) had already done at least 5 Therapeutic Patient Education (TPE) sessions. Also, 81.0% (158/195) were members of a support group (Table 1).

**Table 1:** characteristics of the study population, Mother and Child Center in Yaounde, August to October 2018

Characteristics	
Mean age	16.8 ± 1.5 years
Female/Male sex-ratio	4/3
**Non-adherent**	**n (%)**
Quantitative and qualitative non-adherence to ART	80 (41.0)
Quantitative non-adherence to ART	66 (33.8)
Qualitative non-adherence to ART	14 (7.2)
**Sociodemographic**	**n (%)**
Living out of Yaounde	32 (16.4)
Not schooling	18 (9.2)
Primary school	18 (9.2)
Sexually active	42 (21.5)
Taking alcohol	57 (29.2)
**Clinicobiologic**	**n (%)**
Orphans of both parents	41 (21.0)
Vertical transmission	176 (90.2)
Asymptomatic participants	182 (93.3)
Defaulters	41 (21.0)
Second line regimen	52 (26.7)
Once-a-day medication	181 (92.8)
On Efavirenz regimen	142 (72.8)
CD4 ≤ 500 cells/mm3	82 (42.1)
**Therapeutic and psychosocial strategies**	**n (%)**
HIV status disclosed after 13 years	94 (48.2)
Disclosure of HIV status by health personnel	143 (73.3)
Parents/guardians reminded hour for drug intake	27 (13.8)
Transition preparation not yet started	137 (70.3)
At least 5 TPE* sessions done	92 (47.2)
Members of a support group	158 (81.0)

(*): Therapeutic Patient Education

### Main results

**Factors associated with non-adherence to ART in bivariate analysis:** participants living in a house with monthly income > 150 000 FCFA were significantly higher among non-adherent (60.0% versus 41.7% among adherent, p: 0.013; cOR: 2.09; 95% CI: 1.17-3.75) and were 2.09 times more likely to be non-adherent versus those with > 150 000 F CFA. In contrast, no association between adherent and non-adherent was reported regarding age group, sex, residence, level of education, schooling, sexuality and alcohol use (Table 2).

**Table 2:** socio-demographic factors associated with non-adherence to ART in bivariate analysis, Mother and Child Center in Yaounde, August to October 2018

Factors	Adherent (%)	Non-adherent (%)	N (%)	cOR (95%CI)	P-value
**Age group (years)**					
[15-17]	54 (47.0)	36 (45.0)	90 (46.2)	1	
[17-20]	61 (53.0)	44 (55.0)	105 (53.8)	1.08 (0.61-1.93)	0.787
**Sex**					
Male	49 (42.6)	35 (43.7)	84 (43.1)	1	
Female	66 (57.4)	45 (56.3)	111 (56.9)	0.95 (0.53-1.71)	0.874
**Residence**					
Yaounde	93 (80.9)	70 (87.5)	163 (83.6)	1	
Out of Yaounde	22 (19.1)	10 (12.5)	32 (16.4)	0.60 (0.25-1.33)	0.219
**Education level**					
Primary	12 (10.4)	6 (7.5)	18 (9.2)	1	
Secondary and plus	103 (89.6)	74 (92.5)	177 (90.8)	1.43 (0.47-4.88)	0.656
**Monthly income**					
≤ 150 000 F CFA	67 (58.3)	32 (40.0)	99 (50.8)	1	
> 150 000 F CFA	48 (41.7)	48 (60.0)	96 (49.2)	2.09 (1.17-3,75)	0.013*
**Schooling**					
No	11 (9.6)	7 (8.8)	18 (9.2)	1	
Yes	104 (90.4)	73 (91.3)	177 (90.8)	1.10 (0.40-2,98)	0.847
**Sexually active**					
No	89 (77.4)	64 (80.0)	153 (78.5)	1	
Yes	26 (22.6)	16 (20.0)	42 (21.5)	0.90 (0.44-1.83)	0.795
**Taking alcohol**					
Never	86 (74.8)	52 (65.0)	138 (70.8)	1	
≥ 1 time	29 (25.2)	28 (35.0)	57 (29.2)	1.60 (0.85-2.98)	0.141

(1): Reference value for the calculation of OR; (*): P <0.05; (cOR): crude Odds Ratio; (95% CI): 95% Confidence Interval

Participants on ART > 5 years were significantly higher among non-adherent (78.8% versus 59.1% among adherent, p: 0.006; cOR: 2.47; 95% CI: 1.29-4.70) and were 2.47 times more likely to be non-adherent versus those on ART ≤ 5 years. Moreover, participants with detectable VL (VL ≥ 40 copies/ml) were significantly lower among non-adherent (36.2% versus 51.3% among adherent, p: 0.03; cOR: 0.5; 95% CI: 0.30-0.97) and had 1.85 (1/0.54) times less likely to be non-adherent versus those with undetectable VL (VL < 40 copies/ml) (Table 3).

**Table 3:** clinicobiologic factors associated with non-adherence to ART in bivariate analysis, Mother and Child Center in Yaounde, August to October 2018

Factors	Adherent (%)	Non-adherent (%)	N (%)	cOR (95%CI)	P-value
**Orphans of both parents**					
No	92 (80.0)	62 (77.5)	154 (79.0)	1	
Yes	23 (20.0)	18 (22.5)	41 (21.0)	1.16 (0.57-2.33)	0.674
**Transmission**					
Vertical	102 (88.7)	74 (92.5)	176 (90.3)	1	
Horizontal	13 (11.3)	6 (7.5)	19 (9.7)	0.90 (0.53-1.49)	0.669
**WHO stage**					
Stage 1	105 (91.3)	77 (96.3)	182 (93.3)	1	
Other stages	10 (8.7)	3 (3.7)	13 (6.7)	0.42 (0.13-1.32)	0.173
**Defaulters**					
No	96 (83.5)	58 (72.5)	154 (79.0)	1	
Yes	19 (16.5)	22 (27.5)	41 (21.0)	1.92 (0.95-3.84)	0.067
**Treatment line**					
First line	82 (71.3)	61 (76.3)	143 (73.3)	1	
Second line	33 (28.7)	19 (23.7)	52 (26.7)	1.27 (0.67-2.41)	0.546
**Daily taking**					
Once daily	106 (92.2)	75 (93.8)	181 (92.8)	1	
Twice daily	9 (7.8)	5 (6.2)	14 (7.2)	0.84 (0.52-1.36)	0.476
**Duration on ART**					
≤ 5 years	47 (40.9)	17 (21.2)	64 (32.8)	1	
> 5 years	68 (59.1)	63 (78.8)	131 (67.2)	2.47 (1.29-4.70)	0.006*
**Efavirenz regimen**					
No	35 (30.4)	18 (22.5)	53 (27.2)	1	
Yes	80 (69.6)	62 (77.5)	142 (72.8)	1.51 (0.78-2.91)	0.222
**HIV Viral Load**					
< 40 copies/ml	56 (48.7)	51 (63.8)	107 (54.9)	1	
≥ 40 copies/ml	59 (51.3)	29 (36.2)	88 (45.1)	0.54 (0.30-0.97)	0.039*
**CD4 count**					
≤ 500 c/mm^3^	43 (37.4)	39 (48.8)	82 (42.1)	1	
> 500 c/mm^3^	72 (62.6)	41 (51.2)	113 (57.9)	0.63 (0.35-1.12)	0.115

(1): Reference value for the calculation of OR; (*): P < 0.05; (cOR): crude Odds Ratio; (95%CI): 95% Confidence Interval

Participants whose HIV disclosure was done by health personnel were significantly higher among non-adherent (81.3% among non-adherent versus 67.8% among adherent, p: 0.039; cOR: 2.06; 95% CI: 1.03-4.08) and where 2.06 times more likely to be non-adherent versus those disclosed by their parent/guardian. Regarding HIV knowledge, participants who scored ≥ 12/15 (10.2%) were significantly higher among adherent (15.7% versus 2.5% among non-adherent, p: 0.003; cOR: 0.14; 95% CI: 0.01-0.62) and had 7.14 (1/0.14) times less chance of being non-adherent versus those with < 12/15 (Table 4).

**Table 4:** enhance adherence factors associated with non-adherence to ART in bivariate analysis, Mother and Child Center in Yaounde, August to October 2018

Factors	Adherent (%)	Non-adherent (%)	N (%)	cOR (95%CI)	P-value
**Age at disclosure**					
≤ 13 ans	64 (55.7)	37 (46.2)	101 (51.8)	1	
> 13 ans	51 (44.3)	43 (53.8)	94 (48.2)	1.45 (0.82-2.59)	0.197
**Disclosed by**					
Parent/guardian	37 (32.2)	15 (18.7)	52 (26.7)	1	
Health personnel	78 (67.8)	65 (81.3)	143 (73.3)	2.06 (1.03-4.08)	0.039*
**Hour for ART intake**					
Parent do not remind	97 (84.3)	71 (88.8)	168 (86.2)	1	
Parent do remind	18 (15.7)	9 (11.2)	27 (13.8)	0.73 (0.42-1.27)	0.269
**Transition process**					
Preparation not started	77 (67.0)	60 (75.0)	137 (70.3)	1	
Preparation started	38 (33.0)	20 (25.0)	58 (29.7)	0.68 (0.35-1.28)	0.228
TPE* sessions					
< 5 sessions	63 (54.8)	40 (50.0)	103 (52.8)	1	
≥ 5 sessions	52 (45.2)	40 (50.0)	92 (47.2)	1.21 (0.65-2.24)	0.609
**Support group**					
No	25 (21.7)	12 (15.0)	37 (19.0)	1	
Yes	90 (78.3)	68 (85.0)	158 (81.0)	1.57 (0.73-3.36)	0.240
**HIV Knowledge**					
< 12/15	97 (84.3)	78 (97.5)	175 (89.8)	1	
≥ 12/15	18 (15.7)	2 (2.5)	20 (10.2)	0.14 (0.01-0.62)	0.003*

(1): Reference value for the calculation of OR; (*): P < 0.05; (cOR): crude Odds Ratio; (95%CI): 95% Confidence Interval

**Factors associated with non-adherence to ART in multivariate analysis:** non-adherence to ART was associated negatively with defaulters (aOR: 2.56; 95% CI: 1.12-5.82) and duration on ART > 5 years (aOR: 2.33; 95% CI: 1.08-5.00), but positively with HIV VL ≥ 40 copies/ml (aOR: 0.42; 95% CI: 0.21-0.83) (Table 5).

**Table 5:** factors associated with non-adherence to ART in multivariate analysis, Mother and Child Center in Yaounde, August to October 2018

Factors	cOR (95%CI)	aOR (95%CI)
Household monthly income > 150 000 F CFA	2.09* (1.17-3.75)	1.90 (0.98-3.68)
Defaulters	1.92 (0.95-3.84)	2.56** (1.12-5.82)
Duration on ART > 5 years	2.47* (1.29-4.70)	2.33** (1.08-5.00)
Disclosure of HIV status by health personnel	2.06* (1.03-4.08)	1.96 (0.88-4.32)
Good HIV Knowledge (≥ 12/15)	0.14* (0.01-0.62)	0.64 (0.36-1.13)
Detectable HIV viral load (≥ 40 copies/ml)	0.54* (0.30-0.97)	0.42** (0.21-0.83)

(*): Significant cOR; (**): Significant aOR; (aOR): adjusted Odds Ratio; (cOR): crude Odds Ratio; (95%CI): 95% Confidence Interval

## Discussion

**Key results:** the proportion of non-adherence to ART among adolescents aged 15 to 19 years was 41.0% (33.8% and 7.2% following quantitative and qualitative measure respectively). This non-adherence was associated with monthly income household > 150 000 F CFA, HIV disclosure by health personnel, duration on ART > 5 years, HIV knowledge ≥ 12/15, defaulters and HIV VL ≥ 40 copies/ml.

**Limitations:** the main limitation of this study was the measure of non-adherence through a self-reported questionnaire. This method is not the most sensitive for identifying non-adherent, due to possible memory bias [2, 11, 13]. To limit this bias, non-adherence was assessed during the 3 days preceding inclusion. Prior to questionnaire administration, participants were reminded that the difficulties in adherence are normal and comprehensive. This standard questionnaire was used to maximize reliability [14]. Another limitation included the cross-sectional design, which gives limited room to monitor adherence-level overtime [7].

**Interpretation:** the proportion of non-adherence in this study was lower than performance observed in other studies where adherence was measured through pill count and VL [8, 11, 13, 17]. This low proportion might be attributed to the qualitative measure, which revealed only 7.2% of non-adherent. Table 2 highlighted a high proportion of non-adherent among adolescents aged 17-19 years, especially girls. This observation confirms female sex as a vulnerable population when fighting against HIV/AIDS [18].

Factors identified in the literature such as duration on ART [14] were also highlighted in this study. Indeed, duration on ART > 5 years was correlated with non- adherence. Regarding VL, participants with VL ≥ 50 copies/ml were the most adherent unlike previous studies [14]. Following national guidelines for HIV care in Cameroon, adolescents with VL ≥ 50 copies/ml are followed-up monthly, while those with VL < 50 copies/ml are followed-up quarterly. Moreover, the rate of non-adherence was high among participants whose HIV-status was disclosed by medical personnel. This result confirms that parents/guardians play an important role in the disclosure process [19]. Also, having poor knowledge on HIV/AIDS was associated with non-adherence like in previous studies [14].

HIV disclosure by health personnel was not associated with non-adherence. This finding indicates that there are potential factors in multivariate analysis that influence negatively adherence to ART. The proportion of defaulters was high among non-adherent. This trend was not significant in bivariate analysis, but became significant in multivariate model. In contrast, protective factors such as good knowledge on HIV/AIDS and detectable VL remained unchanged in multivariate analysis. These results confirm the interest of multivariate analysis that takes into account interactions of potential confounding factors.

**External validity:** this study used a simple random sampling and included about 3 out of 4 adolescents from the target population. Hence, the findings reported can be generalized among HIV-infected adolescents aged 15 to 19 years and who are followed-up at this referral pediatric center. Nevertheless, with the monocentric site of this study, it is not possible to draw conclusions on other referral pediatric centers in Cameroon.

## Conclusion

At the MCC in Yaounde, about 4 out of 10 adolescents aged 15-19 years on ART are non-adherent, driven by missing dosage of drug intake. Strategies for enhanced adherence for late age adolescents are therefore warranted, by prioritizing interventions on defaulters and duration on ART of greater than 5 years, while adolescents with detectable VL constitute a target with high adherence. Importantly, a combination method towards adherence assessment would be more productive in resource-limited settings. Such approach might contribute to long-term therapeutic success and smooth transition from pediatric to adult care in this specific population.

### What is known about this topic

- Assessing non-adherence to antiretroviral therapy (ART) using quantitative methods is the gold standard;
- Adolescents living with HIV remain a high-risk group for non-adherence to ART.

### What this study adds

- The first original finding in this study was the positive association between detectable HIV viral load (VL ≥ 40 copies/ml) and non-adherence to ART;
- The second original finding revealed was the duration on ART, particularly > 5 years, which appeared as a risk factor of non-adherence.
